# Coordination Compounds of Cu, Zn, and Ni with Dicarboxylic Acids and N Donor Ligands, and Their Biological Activity: A Review

**DOI:** 10.3390/molecules28031445

**Published:** 2023-02-02

**Authors:** Ivana Loubalová, Pavel Kopel

**Affiliations:** Department of Inorganic Chemistry, Faculty of Science, Palacky University, 17. listopadu 12, 779 00 Olomouc, Czech Republic

**Keywords:** dicarboxylate complex, copper, zinc, nickel, mixed ligand complexes, antibacterial, anticancer

## Abstract

Complexes of carboxylic acids are very often studied due to their interesting structural, spectral, and magnetic properties. This review is focused on complexes of four dicarboxylic acids, namely, 2,2′-thiodioacetic, 3,3′-thiodipropionic, 3,3′-dithiodipropionic, and fumaric acid. Many of the complexes were characterized by single crystal X-ray analyses. Without the analyses, it is very difficult to predict the coordination mode of carboxylate groups or nitrogen ligands on central atoms. Thus, structural properties are also discussed, as well as potential applications.

## 1. Introduction

Coordination compounds containing carboxylates and nitrogen donor ligands are of continuous interest to many researchers. Many papers have been published on the topic and structural, magnetic, spectral, and catalytic properties, and other interesting properties have been revealed [1,2,3]. There are plenty of complexes with carboxylates and N donor ligands that were studied for their magnetic properties [4,5,6,7,8]. Very important compounds formed by carboxylic acids are metal–organic frameworks (MOFs). These were mostly applied for their physical properties but they were also studied as antibacterial agents or drug transporters [9,10,11,12,13,14]. Zinc carboxylates can be utilized as precursors for zinc oxide nanoparticles and are studied for their biological activities [15,16,17].

Copper has been used since ancient times for its therapeutic effects. In ancient Egypt, copper oxide was used with honey or rose oil to disinfect wounds and deworm. In ancient Persia, copper oxide and copper hydroxide-carbonate were applied in ophthalmology [18], while the Aztecs used the same copper compounds to treat skin diseases. In 1761, the antifungicidal effects of copper sulfate were noted. It was further applied from the 1880s in a mixture with sodium carbonate to spray grapes against mold. In 1867, the effects of copper on the immune system were observed in France, where people who worked in copper mines did not fall ill with cholera. The further therapeutic potential was noted in 1939 in Finnish copper mines where miners did not suffer from rheumatic diseases [18]. Nowadays, copper compounds are being prepared for potential antibacterial, antifungicidal, and antiviral effects, but this is not the only direction. The research is also focused on antitumor activity, such as with Casiopeinas^®^ [19].

Zinc in the form of zinc oxide was used in ancient Egypt and India in skin creams, and later zinc oxide was used to stop bleeding [20]. However, other compounds also found use in medicine: zinc chloride for its antiseptic effects, and zinc sulfate to induce vomiting. Since the 1930s, zinc pyrithione has been used for the topical treatment of skin or hair damaged by fungal or bacterial infection. Various coordinating zinc compounds are now being studied for various applications in radioprotection, tumor photosensitizers, antidiabetic agents, anticonvulsants, anti-inflammatory agents, and agents with antimicrobial, antioxidant, and antitumor/antiproliferative properties [21].

Nickel, unlike copper or zinc compounds, has been used in medicine in the past for its therapeutic effects. However, in the previous decades, coordination compounds and nickel nanoparticles have had potential medical applications. The compounds were examined for their antimicrobial, antifungicidal, and antitumor properties [22,23].

**Scheme 1:**
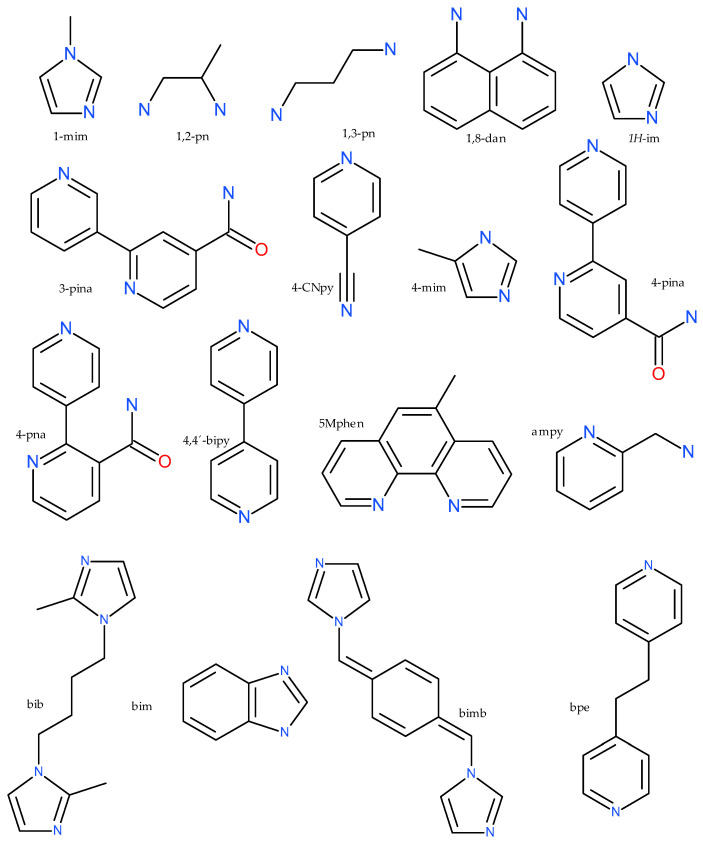

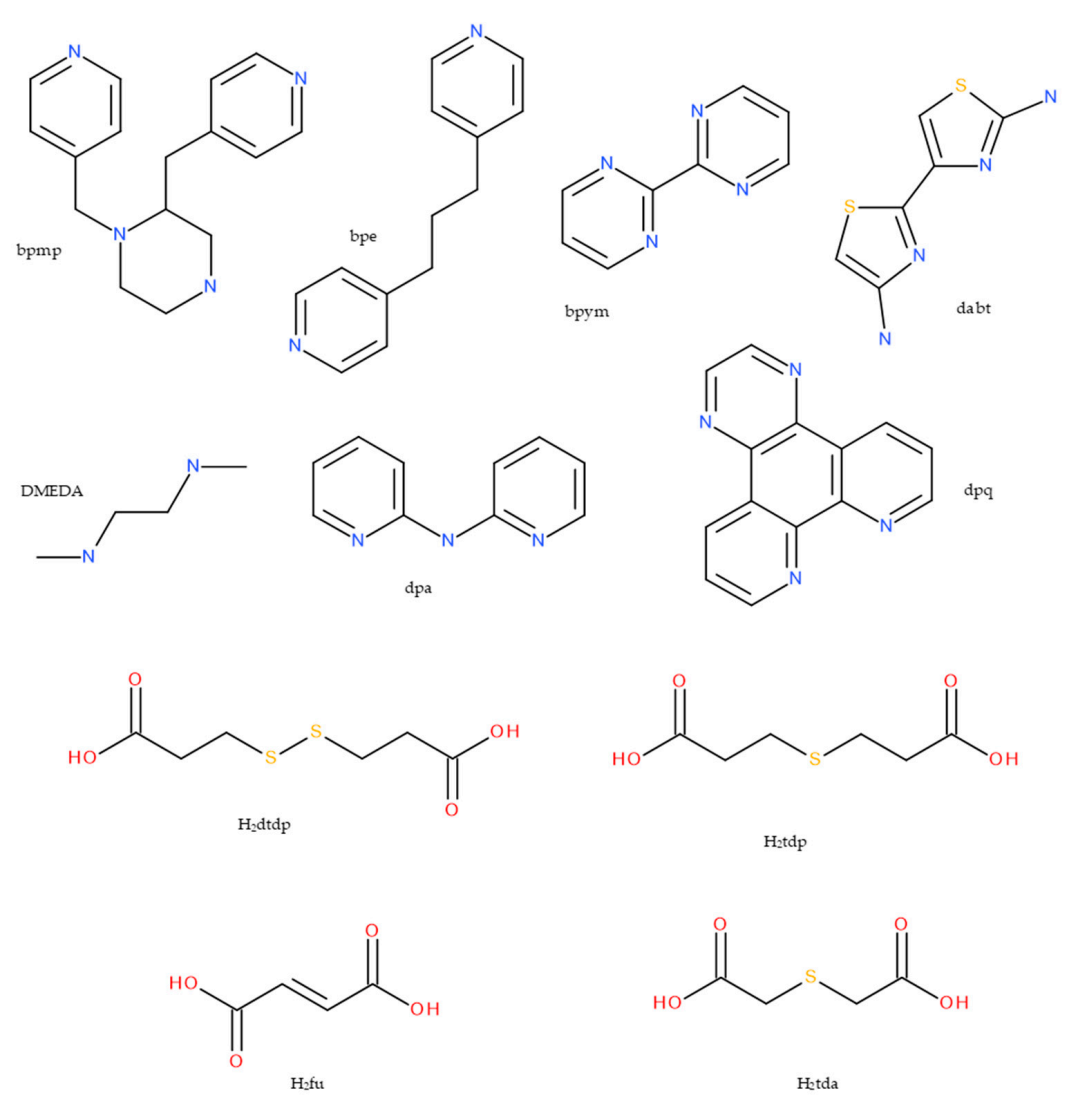

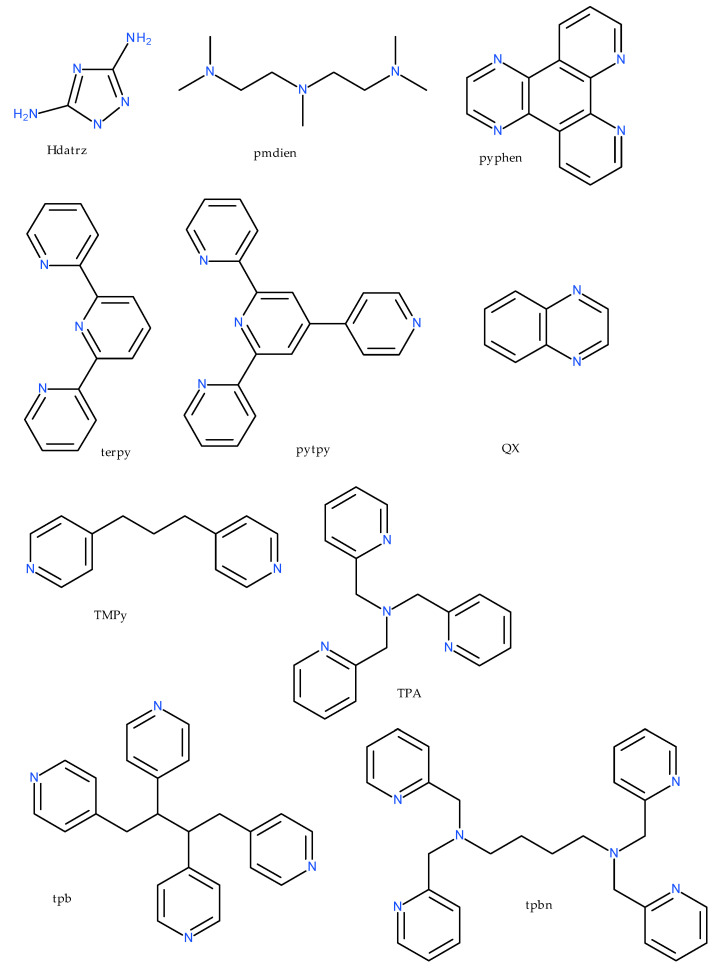

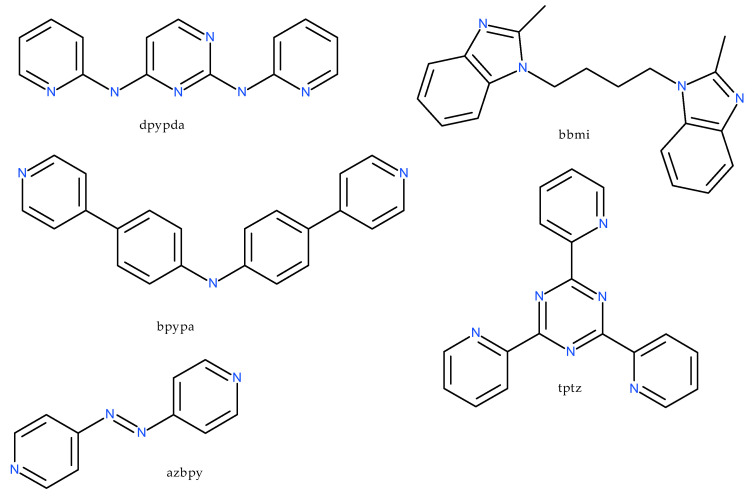
Ligands discussed in the review: 1-mim—1-methylimidazole; 1,2-pn—1,2-propanediamine; 1,3-pn—1,3-propanediamine; 1,8-dan-1,8-diaminonaphthalene; *1H-*im*—1H-*imidazole; 3-pina—3-pyridylisonicotinamide; 4-CNpy—4-cyanopyridine; 4-mim—4-methylimidazole; 4-pina—4-pyridylisonicotinamide; 4-pna—4-pyridylnicotinamide; 4,4′-bipy—4,4′-bipyridine; 5Mphen—5-methyl-1,10-phenanthroline; ampy—2-(aminomethyl)-pyridine; bib—1,4-bis(2-methyl-imidazol-1-yl)butane; bim—benzimidazole; bimb—1,4-bis(imidazol-1-yl-methylene)benzene; bipy—2,2′-bipyridyl; bpe—*trans-1*,2-bis(4-pyridyl)ethane; bpmp—bis(4-pyridylmethyl)piperazine; bpp—1,3-bis(4-pyridyl)propane; bpypa—4,4′-bis(4-pyridyl)diphenylamine; bpym—2,2′-bipyrimidine; dabt—2,2′-diamino-4,4′-bi-1,3-thiazole; DMEDA—*N*,*N′-*dimethylethane-1,2-diamine; dpa—di-2-pyridylamine; dpq—dipyrido[3,2-d:2′3′-f]quinoxaline; H_2_dtdp—3,3′-dithiodipropionic acid; H_2_fu—fumaric acid; H_2_tda—2,2′-thiodiacetic acid; H_2_tdp—3,3′-thiodipropionic acid; datrz—3,5-diamino-1,2,4-triazole; pmdien *N*,*N*,*N*,*N′*,*N″*,*N‴*-pentamethyldiethylenetriamine; pyphen—pyrazino[2,3-f][1,10]phenanthroline; terpy—2,2′:6′,2″-terpyridyl; pytpy—4′-(4-pyridyl)3,2′:6′,3′′-terpyridine; QX—quinoxaline; TMPy—4,4-trimethylenedipyridine; TPA—tris(2-pyridylmethyl)amine; tpb—1,2,3,4-tetra-(4-pyridyl)-butane; tpbn—*N*,*N′*,*N*″,*N‴-*tetrakis(2-pyridylmethyl)-1,4-diaminobutane; dpypda—*N^2^,N^4^*-di(pyridin-2-yl)pyrimidine-2,4-diamine; bbmi—1,1′-(1,4-butanediyl)bis(2-methylbenzimidazole; bpypa—4,4′-bis(4-pyridyl)diphenylamine; tptz—2,4,6-tris(2-pyridyl)-s-triazine; azbipy—4,4′-azobipyridine.

The four dicarboxylic acids were chosen because thiodiacetic acid and fumaric acid are naturally occurring acids, and thiodipropionic, and dithiodipropionic acids are analogous to thiodiacetic acid. Thiodiacetic acid is a metabolite of cysteine which can occur in the human body. Fumaric acid is very often used in the form of iron fumarate as an iron supplement to treat iron deficiency in humans. Thiodipropionic, and dithiodipropionic acids contain sulfur atoms in their structure, similarly to thiodiacetic acid, and sulfur in compounds can increase biological activity. Most of the ligands appearing in this review are depicted in Scheme 1.

## 2. Biologically Active Copper, Zinc, and Nickel Coordination Compounds

### 2.1. Copper Coordination Compounds

Copper coordination compounds have the potential to be biomedically applied due to the essential nature of copper, the variety of stereochemistry, the ability to change the oxidation state, or the relatively low toxicity. Copper interacts with DNA molecules and proteins. Complexes are synthesized that inhibit or inactivate enzymes or are synthesized for biological activity such as anticancer, antimicrobial, and anti-inflammatory activity [24,25]. The complication in bioapplications of copper complexes lies in their lower stability in water and acidic environments and there is also an issue with their water solubility and lipophilicity.

#### 2.1.1. Antitumor Properties of Copper Coordination Compounds

Since the 1960s, studies on the development of the antitumor activity of copper(II) compounds have been extensive, as observed for compounds with thiosemicarbazones (L^1^), whose mechanism of action is based either on the inhibition of certain biological processes (DNA synthesis, mitochondrial respiration) or the inhibition of ribonucleotide diphosphate reductase or the activation of processes leading to apoptosis [26]. These processes are inhibited in such a way that thiosemicarbazone copper complexes react with thiols (RSH) (glutathione, 2-mercaptoethanol) in the intracellular space to form copper intermediates, which subsequently react with molecular oxygen to form ROS [27]:

Cu^2+^ L^1^*_m_* + RSH → RS-Cu^1+^ L^1^*_m_*_− *x*_ + H^+^

2RS-Cu^1+^ L^1^*_m_*_− *x*_ + O_2_ → 2Cu^2+^ L^1^*_m_* + RSSR + O_2_^•−^.

Reactive oxygen species damage cell membranes and DNA molecules or interact between ROS and protein molecules. A copper complex with thiophene-2-carbaldehyde-thiosemicarbazone has demonstrated in vivo activity against erythroleukemia and melanoma [27]. A Cu(II) complex with diacetyl-bis(*N4*-methylthiosemicarbazone) has been shown to have in vitro and in vivo activity in the HeLa cell line as well as in mice [28].

A group of complexes with imidazole, benzimidazole, pyrazole, and triazole ligands in different phases of clinical trials have high cytotoxicity against tongue cancer, skin malignancy, lung and stomach cancer or cervical and prostate cancer [29].

**Scheme 2:**
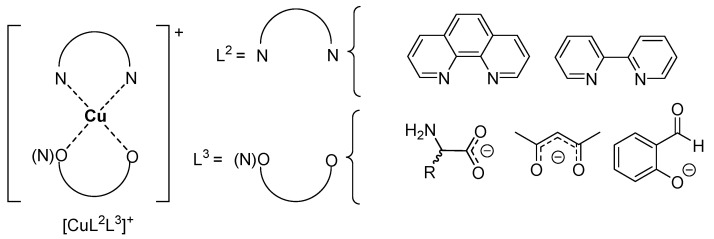
Structural patterns of Casiopeinas representatives^®^ [31].

Complexes of Cu(II) with phenanthroline and bipyridine are able to cause the formation of ROS and induce DNA and RNA degradation. The ligand, phenanthroline, intercalates into the DNA structure and copper induces the formation of hydroxyl radicals in situ, causing DNA degradation [30]. This group includes complexes of copper and phenanthroline or bipyridine with other ligands, called Casiopeinas^®^ (Scheme 2), of which there are several variants [31].

#### 2.1.2. Antibacterial and Antifungicidal Properties of Copper Coordination Compounds

Among the coordination compounds of copper(II) that have antibacterial effects are those that have Schiff bases attached in their structure. Representatives of these complexes are numerous, some being not only effective against bacteria but also against fungi. An example of a coordinated Schiff base is methyl-2-pyridylketonesemicarbazone, where the Cu(II) complex with this ligand is effective against *Escherichia coli*, *Bacillus subtiilis*, *Erwinia catovora*, and *Staphylococcus aureus*, and antifungicidal activity has also been observed (*Candida kefyr*, *Candida krusei*, *Aspergillus niger*) [23].

### 2.2. Zinc Coordination Compounds

Zinc in biological systems is characterized by certain properties, which are also related to its importance. Zn^2+^ in biological systems is not subject to redox processes as it is a strong Lewis acid, and because it has a d^10^ configuration, it is diamagnetic. It exists in various coordination polyhedra (tetrahedron, pyramid, trigonal bipyramid, octahedron), i.e., the coordination numbers are 4, 5, and 6, but it is subject to the rapid exchange of coordinated ligands. Ligands are *N*, *O* or *S* donors, such as histidine, glutamate, aspartate, and cysteine. In some cases, they are dimeric and polymeric complexes.

Zinc is an essential element, indispensable in many processes, where its functions can be divided into circuits where it affects reproduction, the immune system, the sensory and nervous system and is part of proteins and enzymes, as well as having antidiabetic activity (it is part of insulin) [32]. It also inhibits the oxidative damage that is associated with diabetes. Zinc chloride has been administered orally in the last century, but the bioavailability of zinc in this preparation is low [33]. In 2001, the first zinc complexes with antidiabetic properties were discovered (e.g., bis(maltolato) zinc complex, bis(2-aminoethylpyridinato) zinc complex) [32]. In clinical trials, antidiabetic effects and insulin mimetic activity were found for Zn(II) complexes with coordinated ligands, examples of which are cyclo(His-Pro), dithiocarbamate, bis(thioallixin-*N*-methyl), and bis(pyrrolidine-*N*-dithiocarbamate) [33].

Photodynamic cancer therapy is another direction in which zinc complexes have potential applications. Photoexcitation drugs cleave the DNA molecule, either in an aerobic or anaerobic reaction under hypoxic conditions. In a photoexcitation agent, photoexcitation is induced by red light (in the range of 600 to 800 nm, for better penetration through the skin). These forms cause oxidative damage to cancer cells, leading to apoptosis (by a mitochondrial pathway or by activation of death receptors), necrosis, and autophagy. Photosensitive systems (e.g., phthalocyanines, porphyrins, and naphthalocyanines) are coordinated to the central atom in the complexes [34,35].

Zinc can be used as a catalyst in the hydrolysis and cleavage of DNA, hence its possible anticancer activity. A number of coordination compounds of zinc with *N* donor ligands are being studied for cytotoxic activity. It is the complexes with quinoline derivatives that give rise to ROS. Complexes with bipyridine and phenanthroline intercalate into the DNA structure or coordinate to the helix groove. They have been reported to have activity in HeLa cell lines with lower cytotoxicity compared to cisplatin for healthy cells. Their co-ligands tend to be, for example, iminodiacetate, 2,6-pyridine dicarboxylic acid. Complexes of Zn(II) with terpyridines and other pyridine derivatives have the ability to intercalate into DNA. Moreover, photoluminescent properties have been observed for these complexes, which could find application as fluorescent materials or biosensors. Zinc complexes as biosensors interfere with interactions between oncogenic and other proteins, fluorescently sense enzyme activity, or label proteins. Examples of coordinated ligands are dipicolylamine or bis(dipicolylamine), which are labeled with anthracene, and bipyridine. These biosensors may find application in the treatment of cancer or HIV [36].

Zinc compounds are used as non-steroidal analgesics, anti-inflammatory drugs, or antipyretics, where carboxylic acid derivatives (anthranilic, salicylic, and others) are coordinated to the central atom and *N* and *N,N* donor co-ligands are pyridine and its derivatives, imidazole derivatives, bipyridine, phenanthroline, and others.

### 2.3. Nickel Coordination Compounds

In vitro antitumor activity was demonstrated in a human breast cancer cell line for Ni(II) complexes with *o-*naphthoquinonthiosemicarbazone and also with naphthoquinonesemicarbazone [37].

### 2.4. Coordination Compounds of Cu, Zn, and Ni with Dicarboxylic Acids and N Donor Ligands

In nickel, copper, and zinc complexes with dicarboxylic acids and *N* donor ligands, the donor set can be described as *N_x_O_y_S_z_*, where X = 1–4, Y = 1–4, Z = 0–1. The coordination numbers for the central atoms of nickel, copper, and zinc are different. In the case of copper, the coordination numbers are 5 and 6, and rarely 4, and the shape of the coordination polyhedron is a square pyramid, which can sometimes be deformed, or it is a trigonal bipyramid. The shape of the coordinating number 6 is that of a distorted octahedron, and more rarely, a square-planar arrangement is found for number 4. The deformation of the octahedron of copper complexes is both in the sense of deviation from the direction and because of the Jahn–Teller effect [38]. For nickel, there are octahedron-shaped (deformed octahedron) complexes, but also square bipyramids for coordination number 6 [39]. For coordination compounds of nickel with dicarboxylic acids and *N* donor ligands, coordination number 6 is expected, and the coordination polyhedron corresponds to an octahedron (deformed octahedron) [40]. Very rarely, a complex is found where the shape is a square bipyramid [41]. Equally infrequently, the coordinating polyhedron is a square pyramid, i.e., coordination number 5 [42]. Biological systems are characterized by specific properties, which are also related to their structure.

### 2.5. Dicarboxylic Acids

**Scheme 3:**
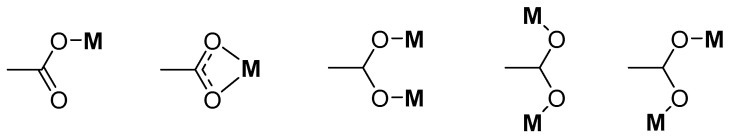
Possible variants of coordination by one carboxylate group (monodentate, chelation, *syn-syn* coordination, *anti-anti* coordination, *anti-syn* coordination).

**Scheme 4:**
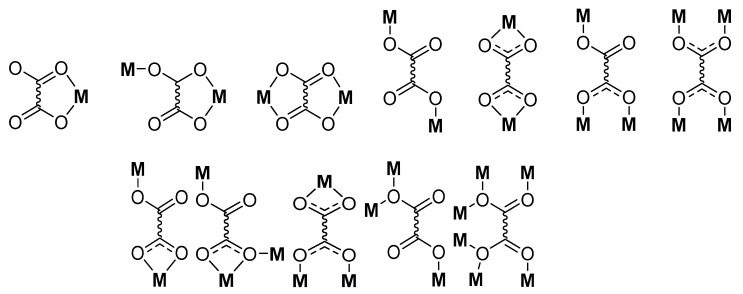
Possible variants of coordination modes of dicarboxylates [45].

The dicarboxylic acids, respectively, 2,2′-thiodiacetic acid, 3,3′-thiodipropionic acid, 3,3′-dithiodipropionic acid, and fumaric acid, can coordinate to the central atom via two carboxyl groups or a sulfur atom from the thioether group and are bidentate as well as monodentate and tridentate [43]. They can be considered as versatile ligands due to their ability to coordinate in bridge form, as chelate ligands, or through carboxyl groups only [44]. Dicarboxylic acids are predominantly in dianionic form. They coordinate in many coordination modes (Scheme 3 and Scheme 4), which accounts for the existence of different nucleation modes of complex compound molecules (they are often mono-, di-, tri-, or tetranuclear) [45].

**Scheme 5:**
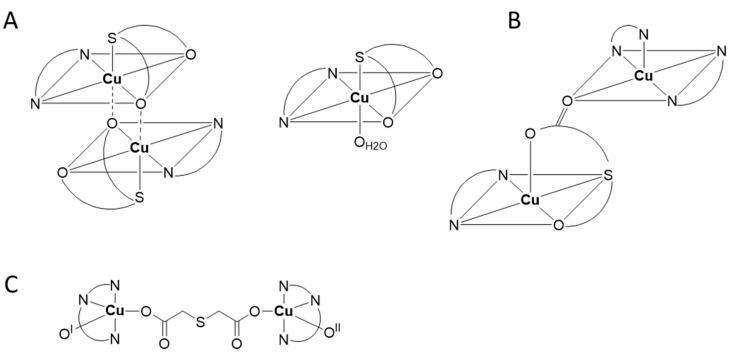
Possible variants of coordination modes of thiodiacetic acid.

In addition to fumaric acid, the previously mentioned acids can have up to five donor atoms. These are four oxygen atoms belonging to two carboxylate groups and a sulfur atom from the thioether group [46]. The coordination modes that have been described for thiodiacetic acid can be divided into several groups [47]:

- The chelating mode *fac-SO*_2_, where the acid acts as a tridentate ligand, exemplified by octahedral complexes of Cu(II), Ni(II), and Zn(II), specifically [Cu(tda)(bipy)(H_2_O)], *fac-*O_2_ + S(apical) (Scheme 5A);
- *fac*-SO_2_ (tridentate) in chelation mode + *μ-*bridging carboxyl group, the first type of this mode is *μ-η^1^* in [Cu(tda)(phen)]_2_·H_2_tda (*fac-*O_2_ + S(apical) + O-monoatomic carboxylate bridging ligand), the second type is *anti,syn-μ-η^1^:η^1^*, where the O,O-diatomic carboxylate bridging group in [(phen)_2_Cu(*μ-*tda)Cu(phen)](NO_3_)_2_·5H_2_O (*fac-*SO + O(apical)) (Scheme 5B), or the third type is *fac-*SO + O(apical) pentadentate (double *anti,syn-μ-η^1^:η^1^*);
- A so-called head-to-tail carboxylate-carboxylate bridge without sulfur atom coordination, where the carboxylate is monodentate (Scheme 5C), for example [Cu_2_(tda)(pmdien)_2_(H_2_O)](ClO_4_)_2_ [47].

For complexes with dicarboxylic acids, which are in the form of a bridge, they mediate interactions between metal centers where the distance between the central atoms of the M—M intradimer is generally greater than 6 Å. Bridges (dicarboxylic acid anions), more precisely their various coordination modes, influence the magnetic behavior, with weak antiferromagnetic or ferromagnetic interactions between the central copper atoms [48]. Zinc complexes are diamagnetic [49,50] and nickel complexes are mostly antiferromagnetic.

Dicarboxylic acid anions in the form of a bridge ligand form 1D, 2D, and 3D polymer networks, in some cases with large cavities, and are so-called metal–organic frameworks (MOFs), which are characterized by thermal stability and durability. Coordination between the central metal atom and the ligand, covalent or non-covalent interactions, or both contribute to the stabilization of these polymers [51]. The non-covalent interactions in these networks are hydrogen bonding [52], where they are often O-H···O interactions, C-H···O interactions [53], and N-H···O interactions [54]. There are cases where 3D networks are stabilized by hydrogen bonds between other atoms, e.g., sulfur atoms of the thioether group. This is a relatively weak intermolecular interaction that is between centrosymmetric sulfur atoms from the thioether. The length of the interaction is considered short because this distance is shorter than the sum of the van der Waals radii. For example, this interaction has been observed for the complexes [Zn(bipy)(tda)(H_2_O)]·4H_2_O and [Cu(bipy)(tda)(H_2_O)]·4H_2_O [55]. Other non-covalent interactions that play a role in the stability of polymer networks are *π*–*π* and C-H–π interactions.

The reaction systems that are used to prepare complexes with aliphatic dicarboxylic acids (2,2′-thiodiacetic acid, 3,3′-thiodipropionic acid, 3,3′-dithiopropionic acid, and fumaric acid) and with *N* donor ligands can be summarized in the form of a general reaction scheme: 

metal precursor—dicarboxylic acid—ligand–solvent.

For copper complexes, Cu(Oac)_2_·H_2_O, [56] Cu(NO_3_)_2_·3H_2_O, [57] Cu(NO_3_)_2_·8H_2_O, [58] Cu(ClO_4_)_2_·6H_2_O [8], CuCl_2_·2H_2_O [59], and Cu_2_(OH)_2_CO_3_ [60]. Zn(NO_3_)_2_·6H_2_O [61], ZnSO_4_·7H_2_O [62], ZnCl_2_ [63], and Zn(Oac)_2_·4H_2_O are used as a zinc precursor [64]. The precursor of nickel is Ni(NO_3_)_2_·6H_2_O [65], NiCl_2_·6H_2_O [40,66], and Ni(Oac)_2_·4H_2_O. Water is most often used as a solvent, as well as methanol, ethanol, [67] DMF [68], THF [69], and ACN. Sometimes the acid solution is neutralized with KOH [70], NaOH [71], and Na_2_CO_3_ [72]. In some cases, a base (trimethylamine, ethylenediamine, piperidine) is used [73]. The nuclearity of complexes depends on the metal-to-ligand stoichiometry, denticity of the ligand and possibilities to chelate, and on reaction conditions. Mononuclear and dinuclear complexes are usually prepared at room temperature or a temperature under 60 °C. With higher temperatures, polymeric complexes are very often obtained. In the case of solvothermal synthesis more complicated structures are obtained and mostly the synthesis leads to metal–organic frameworks.

## 3. Copper, Zinc, and Nickel Dicarboxylate Compounds

### 3.1. Copper

#### 3.1.1. Copper with 2,2′-Thiodiacetic Acid and N Donor Ligands

There are more than a dozen representatives of copper complexes with 2,2′-thiodiacetic acid and *N* donor ligands. Table 1 lists the complexes prepared and structurally characterized to date.

Copper complexes with coordinated 1,10-phenanthroline have been previously studied for their potential biological activity. This activity has been studied for complexes with another ligand, namely the 2,2′-thiodiacetic acid anion [(phen)_2_Cu(*μ-*tda)Cu(phen)] (ClO_4_)_2_·1.5H_2_O and [Cu(phen)(tda)]·2H_2_O. For the mononuclear complex [Cu(phen)(tda)]·2H_2_O, the crystal structure is not stabilized by *π*–*π* interactions, but only by C-H···O interactions. The situation is different for the dinuclear complex [(phen)_2_Cu(*μ-*tda)Cu(phen)](ClO_4_)_2_·1.5H_2_O, where the crystal structure is stabilized not only by *π*–*π* interactions between the centroids of the pyridine and phenyl rings but also by C-H···O hydrogen bonding. Antibacterial activity for both complexes was observed in Gram-positive bacteria (*Bacillus subtilis*, *Staphylococcus aureus*, *Enterococcus faecalis, Escherichia coli*, *Klebsiella pneumoniae*, *Pseudomonas aeruginosa*), the highest in *B. subtilis, S. aureus* and *E. faecalis* [8]. However, when comparing the two complexes, the [Cu(phen)(tda)]·2H_2_O complex is more potent [73]. A similar complex [(phen)_2_Cu(*μ*-tda)Cu(phen)](NO_3_)_2_·5H_2_O has been prepared previously but its biological activity has not been studied.

Another direction aimed at studying biological activity is the attempt to prepare and study structures resembling binuclear metalloenzymes, such as catechol oxidase [76]. Catecholase is a type 3 blue-copper protein [77], whose activity involves the reversible coordination of oxygen molecules at room temperature, which is used to oxidize *o-*diphenols to form *o-*quinones. In 2010, a paper was published by Neuman’s group discussing the synthesis of [Cu(tda)(phen)]_2_·H_2_tda [74], but it was not until 2020 that a paper was published by Ahmad’s group on the re-preparation and subsequent study for the putative and subsequently demonstrated catecholase activity on the substrate 3,5-di-tert-butylcatechol to form 3,5-di-tert-butylquinone. This oxidation of the substrate is accompanied by the reduction of Cu(II) to Cu(I). There are several types of intermolecular interactions in [Cu(tda)(phen)]_2_·H_2_tda: C-H···O, C-H···S, and C-H-π interactions stabilize the 1D structure; the 2D network is stabilized by hydrogen bonds, C-H-π interactions, and *π-π* interactions [76].

**Figure 1:**
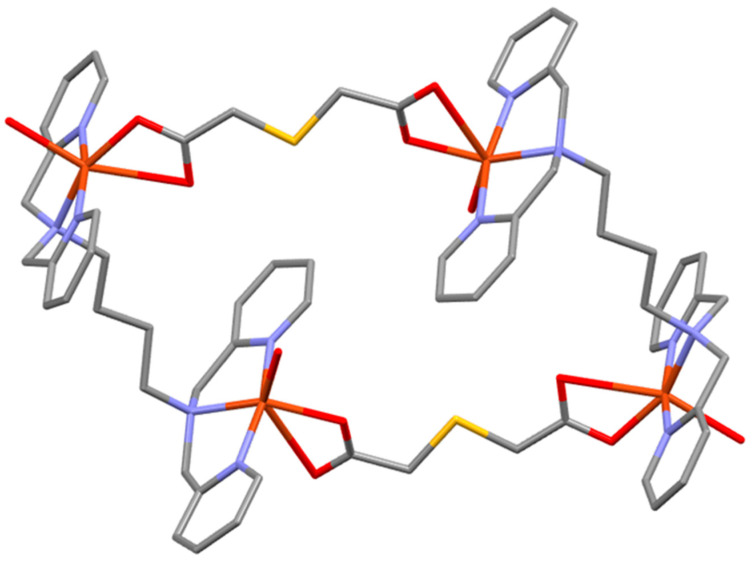
Molecular rectangle structure of the complex [Cu_4_(tpbn)_2_(tda)_2_(H_2_O)_4_](ClO_4_)_4_. (Cu, N, O, S).

The tetranuclear complex [Cu_4_(tpbn)_2_(tda)_2_(H_2_O)_4_](ClO_4_)_4_ (Figure 1) was investigated for adsorption capabilities, which are enabled by the rectangular structure with an internal cavity. This shape is due to the bridges, which are thiodiacetic acid anions. Up to 16 water molecules can be adsorbed into the pores, but water molecules are not the only ones that can be adsorbed; others are CO_2_ molecules, which can be selected from N_2_ molecules [75].

It is obvious that tda can chelate with the S atom coordinated to the copper central atom when monodentate or bidentate N donor ligands are used. When tridentate N ligands are used, binuclear or polynuclear complexes are formed, where the S atom of tda is not involved in coordination. The application of bridging chelating ligand tpbn led to a tetranuclear copper complex with bridging tda.

#### 3.1.2. Copper with 3,3′-Thiodipropionic Acid and N Donor Ligands

There is not a large number of prepared coordination compounds of copper with 3,3′-thiodipropionic acid. Table 2 shows the complexes prepared so far, which have been structurally characterized.

The monodentate thiodipropionic acid anion is the bridging ligand in the complex [Cu_2_(pmdien)_2_(H_2_O)_2_(*μ*-tdp)](ClO_4_)_2_·H_2_O [53]. The presence of hydrogen bonds, O-H···O interactions, causes the formation of a helix-shaped chain, but this type of bonding is not the only one found in this complex. Other hydrogen bonds, C-H···O interactions, allow the existence of a 3D structure by joining adjacent helices through the chloride atoms. The complex is ferromagnetic, where the interaction between the Cu—Cu dimer is probably realized through O-H···O interactions. The substance is characterized by cytocompatibility with mammalian cells (MDA-MB-231 and HBL-100 cell lines), does not show ROS formation, does not affect cell proliferation and growth, and binds to DNA only in minute amounts. In addition, the substance is hemocompatible (no release of hemoglobin on incubation) but induces protein (albumin) aggregation in human plasma. Biocompatibility is another property that appears with this substance, i.e., it does not induce any regulation of p53 either. However, it does induce extracellular matrix breakdown (affects matrix metalloproteinase 1), even in metastatic cancer. Under these assumptions, the expected activity in combination therapy for metastasis is under further study. It stimulates the expression of MT-1/2 and MT-3, leading to the maintenance of cellular metal homeostasis and metal detoxification [53]. 

Some studies have looked at chromotropic properties, where color change takes place depending on chemical or physical conditions. These complex compounds have potential applications as molecular switches, color indicators, and sensors (whether optical, chemical, or thermal). Color changes are usually caused by a change in the coordination polyhedron or by an interaction between the solvent and the complex [79]. Some chemosensors are capable of detecting methanol and ethanol. The rapid detection of methanol in ethanol is crucial as methanol is poisonous and toxic to the human body. Chromotropic complexes are of interest for their speed of detection (colorimetry) compared to the instrumental methods used (luminescence spectroscopy, HPLC, GC/MS) [78]. Two examples are complexes that have the potential to be used as molecular switches, color indicators, or sensors. This is the mononuclear complex [Cu(tdp)(H_2_O)(bim)_3_]·4H_2_O, which was prepared in a mixture of water:ethanol: DMF. If this was not the case, and only water solvents were used. With ethanol, a polymer would have been formed with the formula {[Cu(*μ*_2_-tdp)(bim)_2_]·4H_2_O}*_n_* [79]*_._* It is not only the mode of coordination that differs, with the mononuclear complex having the 3,3′-thiodipropionic acid anion appearing for the first time as a monodentate ligand, whereas in the polymeric complex, the tdp^2−^ is bidentate. Furthermore, the shape of the coordination polyhedron is also different, the former complex having ligands at the vertices of a deformed square pyramid and the latter at the vertices of a deformed octahedron. The color change is affected by the change in solvent or the change in the coordination polyhedron. The solvatochromic change is due to the solvents methanol and dimethylformamide. The former complex changes from blue to light green, the change being due to the transformation in DMF from a deformed square pyramid to a tetrahedron or planar square arrangement. In the supramolecular structure, water molecules are bound in the cavities. If the complex is in methanol, there is an exchange of water molecules to methanol molecules in the cavities, and this is probably the cause of the color change. In the case of {[Cu(*μ*_2_-tdp)(bim)_2_]·4H_2_O}*_n_*, the color change is only in the case of DMF, where a light green color is produced instead of a light blue color, which is due to the formation of the so-called host–guest interaction/non-covalent interaction. However, this is not the only chromotropism that occurs in these two complexes. They are reversibly thermochromic at 65 °C, with the resulting color being green from the original blue that transitions to light blue. This property is due to repeated dehydration and rehydration, which proceed according to the following equations:

[Cu(tdp)(H_2_O)(bim)_3_]·4H_2_O ↔ [Cu(tdp)(H_2_O)(bim)_3_]·H_2_O

{[Cu(*μ*_2_-tdp)(bim)_2_]·4H_2_O}*_n_* ↔ {[Cu(*μ*_2_-tdp)(bim)_2_]·H_2_O}*_n._*

It forms a 1D chain involving a C-H-π interaction that is mediated between the carbon and hydrogen atoms of benzimidazole (C7-H7, C19-H19) and the benzene ring. Hydrogen bonds are also involved in the chain formation; these are between the benzimidazole (N-H) atoms and the tdp^2−^ of the ligand or its carboxylate group. One-dimensional chains are joined into a 2D supramolecular structure by O-H···O type hydrogen bonds; and 3D supramolecular structure is possible due to interactions between benzene rings (Cg2), i.e., *π*–*π* interactions. The coordination is mediated by the oxygen atom of the carboxylate group. The other atoms that are located at the vertices of the coordination polyhedron of the deformed square pyramid are the three nitrogen atoms that belong to the three benzimidazole molecules. One water molecule coordinates at the last site and the other four water molecules are crystal bound.

Another representative that exhibits chromotropism is the blue crystalline substance [Cu_2_(*μ*_2_-tdp)(phen)_4_](NO_3_)_2_·2H_2_O. If Cu(NO_3_)_2_·3H_2_O is used as a copper precursor, the dinuclear [Cu_2_(*μ*_2_-tdp)(phen)_4_](NO_3_)_2_·2H_2_O is formed, but if the copper source Cu(Oac)_2_·H_2_O is used, the resulting coordination compound is the polymer {[Cu(*μ*_3_-tdp)(phen)]·2H_2_O}*_n_* [78]. The shape of the coordination polyhedron in [Cu_2_(*μ*_2_-tdp)(phen)_4_](NO_3_)_2_·2H_2_O is a deformed octahedron due to the Jahn–Teller effect. The 2D structure is stabilized by *π*–*π* interactions (between phenanthroline rings) and C-H···O interactions (between phenanthroline and the oxygen of the carboxylate group). The complex [Cu_2_(*μ*_2_-tdp)(phen)_4_](NO_3_)_2_·2H_2_O is characterized at 140 °C by thermochromism, which is irreversible and is a transition from blue to green. The polymer {[Cu(*μ*_3_-tdp)(phen)]·2H_2_O}*_n_* does not exhibit these properties. It is formed by 20-membered rings due to the presence of a thiodipropionic acid anion bridge. Furthermore, a Cu_2_O_2_ ring appears in the structure, which forms a 1D polymer network with the 20-membered ring. The *π*–*π* interactions and C-H–π interactions that are between the phenanthroline rings reinforce the 2D and 3D crosslinking, in addition to which hydrogen bonds O-H···O are involved, which are mediated by water molecules and oxygen atoms belonging to the carboxylate group [78].

[Cu(*μ*_3_-tdp)(im)_2_]*_n_*, {[Cu(*μ*_3_-tdp)(1-mim)_2_]·0,5H_2_O}*_n_*, and {[Cu_2_(*μ*_3_-tdp)_2_(4-mim)_4_]·H_2_O}*_n_* are coordination polymers that form 1D, 2D, and 3D networks [46]. The 1D helical polymer in [Cu(*μ*_3_-tdp)(im)_2_]*_n_* is generated by two bridges of the thiodipropionic acid anion and forms a 20-membered ring together with Cu_2_O_2_. The 2D supramolecular network is mediated by *π*–*π* interactions and N-H···O hydrogen bonds, and intermolecular C-H···O hydrogen bonds form the 3D network. The 20-membered cycle and the Cu_2_O_2_ also appear in the complex {[Cu(*μ*_3_-tdp)(1-mim)_2_]·0.5H_2_O}*_n_*. The 2D polymer network exists due to the presence of hydrogen bonds, O-H···O interactions, and *π–π* interactions that are between the centroids of 1-methylimidazoles. Another hydrogen bond, the C-H···O interaction, is the reason for the 3D supramolecular network.

While other complexes of copper with 3,3′-thiodipropionic acid show chromotropism or biological activity, the polymeric {[Cu_2_(tdp)_4_(QX)]*_n_*·DMF}*_n_* has catalytic effects in the reduction of 4-nitrophenol [80]. The complex belongs to MOFs and is characterized by the existence of 2.647 nm cavities, which allow the adsorption of nitrogen molecules.

The tdp differs from tda by longer chains. The longer chain seems to prevent tdp from chelating, i.e., S coordination. Only the bridging mode of tdp was proved, and it does not depend on the denticity of N donors. Most copper tdp complexes are polynuclear. The binuclear complex was prepared with tridentate pmdien and copper perchlorate. The perchlorate counter ions are probably the reason for very good solubility.

#### 3.1.3. Copper with 3,3′-Dithiodipropionic Acid and N Donor Ligands

The number of coordination compounds of copper with 3,3′-dithiodipropionic acid and *N* donor ligands is sporadic, and only three have been structurally characterized. The structurally characterized complexes are listed in Table 3 with the indicated donor sets and coordination polyhedra.

**Scheme 6:**
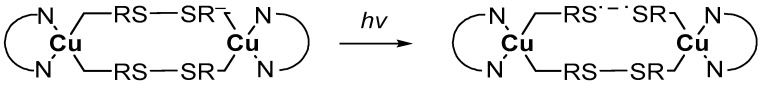
The probable mechanism of photoexcitation leading to S-S bond cleavage [81].

One copper compound with dtdp^2−^ and *N* donor ligand is interesting in that it may act as a photoactive agent that could have applications as a photoexcitation drug in photodynamic therapy (PDT) of cancer. Photoexcitation drugs cleave the DNA molecule, either in an aerobic or anaerobic reaction under hypoxic conditions. In a photoexcitation agent, red light photoexcitation (in the range of 620 to 800 nm, for better penetration through the skin) is induced and reactive oxygen species (ROS) are produced. These forms cause oxidative damage to cancer cells [83]. For the complex [{(phen)Cu}_2_(*μ*-dtdp)_2_]·2H_2_O, where the anion of dithiodipropionic acid and phenanthroline acts as a ligand; photoinduced cleavage of the superhelical winding of the DNA molecule has been observed under UV light (365 nm), red light (647.1 nm) and red light (greater than 750 nm under aerobic and anaerobic conditions). The ligand dtdp^2−^ in the complex acts as a photosensitizer and the phenanthroline in this complex coordinates with the DNA molecule. Photoexcitation cleaves the S-S bond in the dtdp^2−^ ligand to form the anion radical RS^•−^—(Scheme 6) (by transferring an electron from the phenanthroline ring through the central copper atom to the S-S bond), which subsequently cleaves the DNA molecule [81]. The complex was further studied for potential binding to the calf thymus DNA molecule, where the complex coordinates to the large coil. This coordination does not lead to significant changes in the viscosity of the DNA; thus, the complex does not cause changes in the conformation of the DNA. Intercalation was assumed, but only a slight hypochromic shift of the absorption band describing the interactions between the aromatic chromophore and the base pairs of the DNA molecule was observed, and, therefore, this substance is not intercalating in nature. However, when catalases (e.g., SOD) are present, photoinduced DNA cleavage is inhibited provided OH^•^ or O_2_^•−^ are involved in the photoinduced cleavage of DNA molecules (under aerobic conditions) [81]. Antibacterial study of [Cu_2_(*μ*-dtdp)(pmdien)_2_(H_2_O)_2_](ClO_4_)_2_ evaluated against *E. faecalis*, *S. aureus, P. aeruginosa*, *E. coli* bacteria showed MIC 8.44, 8.44, 1.05, and 16.88 mg/L, respectively.

Similarly to tdp, dtdp cannot form a chelate, and the bridging mode is the only way of coordination. When bidentate phen is used in combination with dtdp in 1:1 stoichiometry, a binuclear complex is formed. A binuclear complex similar to that of pmdien and the tdp bridge was prepared in the same way. [Cu_2_(*μ*-dtdp)(pmdien)_2_(H_2_O)_2_](ClO_4_)_2_ is also soluble in water and alcohols.

#### 3.1.4. Copper with Fumaric Acid and N Donor Ligands

Fewer than two dozen copper complexes with fumaric acid and *N* donor ligands were prepared and characterized and are listed in Table 4.

A copper complex with coordinated fumarate {[Cu_2_(L^4^)_2_(fu)]·(H_2_O)·(MeOH)}*_n_*, where HL^4^ is a Schiff base that was prepared by reacting 2-amino-1-butanol with salicylaldehyde has also been studied as a potential metallo-pharmaceutical for its biological activity. The complex shows a bathochromic shift (280 nm) with serum albumin (6 nm with BSA, 7 nm with HSA), and the hypochromic shift (340 nm) in the fluorescence spectrum represents the binding of the complex with serum albumin (3 nm BSA, 2 nm HSA). Electron absorption spectral titration showed the intercalation of the complex into the CT-DNA structure. The complex was further studied for magnetic properties, showing antiferromagnetic interactions between the central atoms in a pseudo-dinuclear model [86].

For [Cu(dpypda)_2_(fu)_2_]*_n_*·8*n*H_2_O, the 1D polymer exists as a zigzag structure whose bridging ligand is fu^2−^ and acts both monodentate and bidentate in one unit. Here, however, one central copper atom is part of a coordination polyhedron in the shape of a trigonal bipyramid, and the other copper atom of a deformed octahedron. Clusters of water molecules (H_2_O)_16_ occur in the gaps of its 3D network [84].

### 3.2. Zinc

#### 3.2.1. Zinc with 2,2′-Thiodiacetic Acid and N Donor Ligands

In order of units, the zinc complexes with 2,2′-thidioacetic acid and *N* donor ligands are also structurally characterized and listed in the following Table 5.

One of the few representatives of coordination compounds of zinc with thiodiacetate and an *N* donor ligand is the coordination polymer [Zn(bib)(tda)]*_n_*, whose 1D chain exists in a helical shape, which is realized via a thiodiacetic acid anion whose carboxylate groups are V-shaped (with an angle of 120°). The ligand 1,4-bis(2-methylimidazol-1-yl)butane is in the *cis* conformation, which is a bridge between zinc atoms [90]. The adjacent zinc atoms are linked by the thiodiacetic acid anion to form the right- and left-handed forms. The angle that is maintained in the helix polymer between the Zn-Zn-Zn atoms is 180°. The polymer helix chain [Zn(tda)]*_n_* is bridged by the ligand bib into a 2D network [90].

The structures of only three zinc complexes with tda were solved. The chelating mode of tda was observed in combination with bipy and phen similar to copper complexes. The bridging mode of tda was found in the polymer complex with bridging N ligand bib.

#### 3.2.2. Zinc with 3,3′-Thiodipropionic Acid and N Donor Ligands

A small number of zinc complexes prepared and structurally characterized so far with 3,3′-thiodipropionic acid and *N* donor ligands, together with their donor set and coordination polyhedra, are listed in Table 6. Complexes with bipyridyl ligands have interesting structures and are described below, along with an additional coordination polymer [61,91].

**Figure 2:**
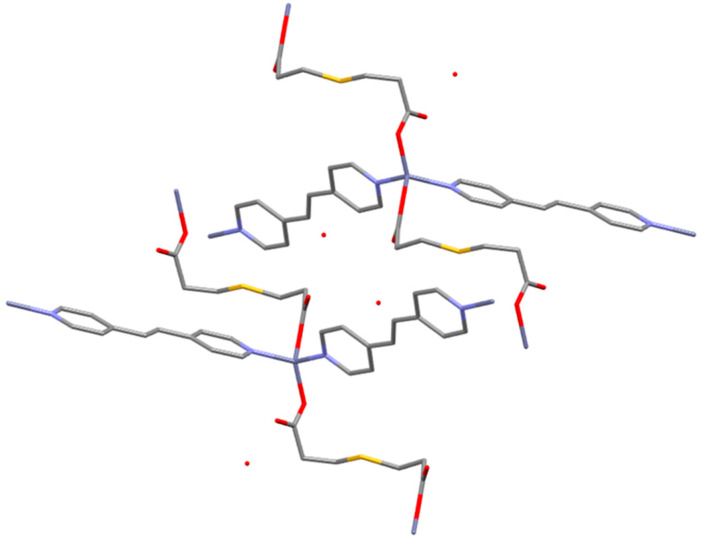
One-dimensional chains linked by *trans*-*1*,2-bis(4-pyridyl)ethylene [61]. (Zn, N, O, S).

Other coordination polymers include three representatives with bipyridyl ligands, *trans-*1,2-bis(4-pyridyl)ethyl (bpe), 1,3-bis(4-pyridyl)propane (bpp), 4,4′-bis(4-pyridyl)diphenylamine) (bpypa), whose formulas are {[Zn(tdp)(bpe)]·2H_2_O}*_n_*, [Zn(tdp)(bpp)]*_n_*, and [Zn(tdp)(bpypa)]*_n_*, where bis-monodentate flexible thiodipropionic acid anion is coordinated. In the case of the {[Zn(tdp)(bpe)]·2H_2_O}*_n_* complex, adjacent zinc atoms are linked by the tdp^2−^ ligand to form a zigzag conformation of the 1D chain. The ligand *trans*-1,2-bis(4-pyridyl)ethylene, where the angle maintained between the two pyridyl rings is 0°, connects the aforementioned 1D chains into 2D layers (Figure 2). The 2D network is further connected by O-H—O type hydrogen bonds, forming a 3D network [61].

The second representative is [Zn(tdp)(bpp)]*_n_*, but here the zigzag conformation of the 1D coordination polymer [Zn(tdp)]*_n_* is coupled to a 2D network involving 1,3-bis(4-pyridyl)propane, where in 1,3-bis(4-pyridyl)propane the two pyridyl cycles form an angle of 77.13°. Invariably, this 1D network is connected by one equivalent network to form a doubly connected 2D network. The supramolecular 3D structure is stabilized by hydrogen bonds, O-H···O, and *π*–*π* interactions [61].

**Figure 3:**
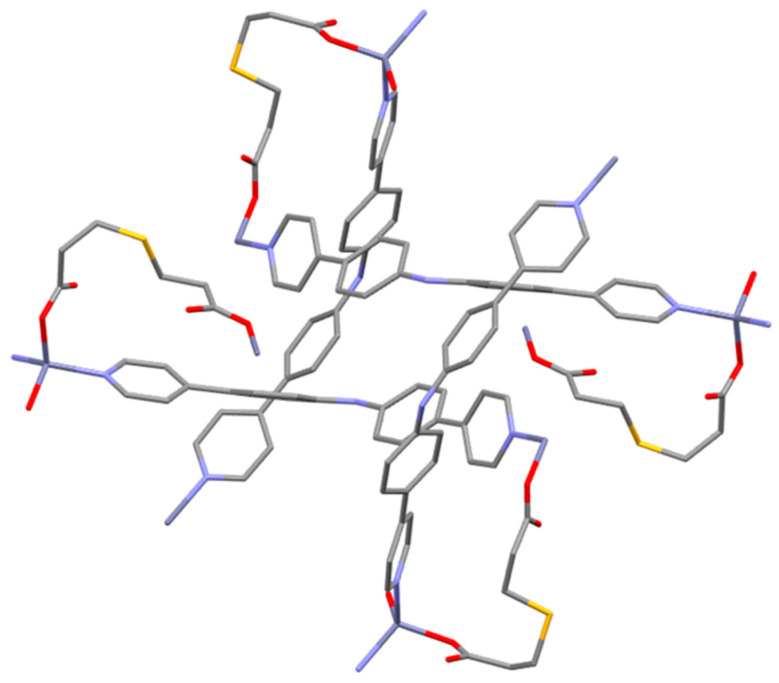
Doubly connected 2D network of the [Zn(tdp)(bpypa)]*_n_* [61]. (Zn, N, O, S).

The third is the [Zn(tdp)(bpypa)]*_n_* complex and here the pyridyl rings of the ligand 4,4′-bis(4-pyridyl)diphenylamine have an angle of 74.08° with respect to each other. The bridge of the thiodipropionic acid anion is the reason for the existence of the zigzag conformation of the 1D polymer. As with the previous two complexes, the bpypa ligand is the bridge between the 1D polymers (Figure 3). The 3D structure is stabilized by *π–π* interactions (between the centroids of the aromatic rings) and N-H···O hydrogen bonds [61].

These three complexes with bipyridyl ligands have been studied for their luminescence properties and their photoluminescence emission maxima are at 541 nm, 418 nm, and 548 nm [61].

[Zn(tdp)_2_(TMPy)_2_]*_n_* is another representative of porous coordination polymers that have adsorption capacity, which is one possible way to remove organic sulfur compounds from diesel fuel [91]. The 2D coordination network adsorbs dibenzothiophene sulfoxide formed by the oxidation of dibenzothiophene by hydrogen peroxide. This reaction is catalyzed by a Cu(II) complex [Cu_2_(BAc)_4_(QX)_2_]. Adsorption is conditioned by the existence of van der Waals and dipole–dipole interactions, *π*–*π* interactions between the aromatic rings of dibenzothiophene sulfoxide and the zinc complex, and the electronegativity of the oxidized and unoxidized forms. Furthermore, the interaction between adsorbent and adsorbate is in the form of charge transfer (from the oxidized organic sulfur compound to the zinc complex) and C-H-π interactions.

#### 3.2.3. Zinc with 3,3′-Dithiodipropionic Acid and N Donor Ligands

In the case of the coordination compounds of zinc with 3,3′-dithiodipropionic acid and *N* donor ligands, no coordination compound was found to be structurally characterized.

One can notice that zinc tdp complexes are polymers with only one exception of the binuclear phen tdp complex. Interestingly, when bipy was used, a polynuclear complex was prepared. It is probably due to the rigidity of phen in contrast to bipy.

#### 3.2.4. Zinc with Fumaric Acid and N Donor Ligands

In the case of coordination compounds of zinc with fumaric acid and *N* donor ligands, there are more representatives that have been prepared and structurally characterized so far than in the case of zinc complexes with other previously mentioned acids. A list of these complexes is given in Table 7.

**Figure 4:**
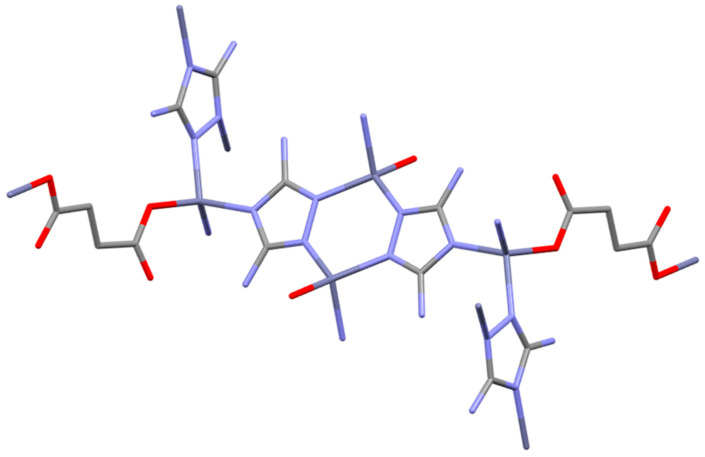
Two-dimensional polymer from units of [Zn_2_(datrz)_2_] [93]. (Zn, N, O).

The complex [Zn(fu)(datrz)_2_]*_n_* is a 3D microporous coordination polymer that forms primitive cubic lattices according to the classification (Figure 4) [93]. The donor atoms are arranged in a tetrahedron shape, with three nitrogen atoms belonging to the three ligands datrz^1–^ and one oxygen atom belonging to the monodentate ligand fumarate. The 2D network of this complex is formed by dinuclear units [Zn_2_(datrz)_2_] (Figure 4), which are connected by bridges of the fumaric acid anion ligand to form the 3D structure (Figure 4). Hydrogen bonds are present between the uncoordinated amino groups of the 3,5-diamino-1,2,4-triazole ligand and the carboxylate groups (oxygen atoms of the fumaric acid anion). The highly thermally stable coordination polymer was studied for its potential adsorption properties, which were shown for N_2_ molecules. Next, adsorption tests were performed for carbon dioxide molecules, with this MOF showing high values [93], better than the known MOFs-5 and MOF-177 [106].

**Figure 5:**
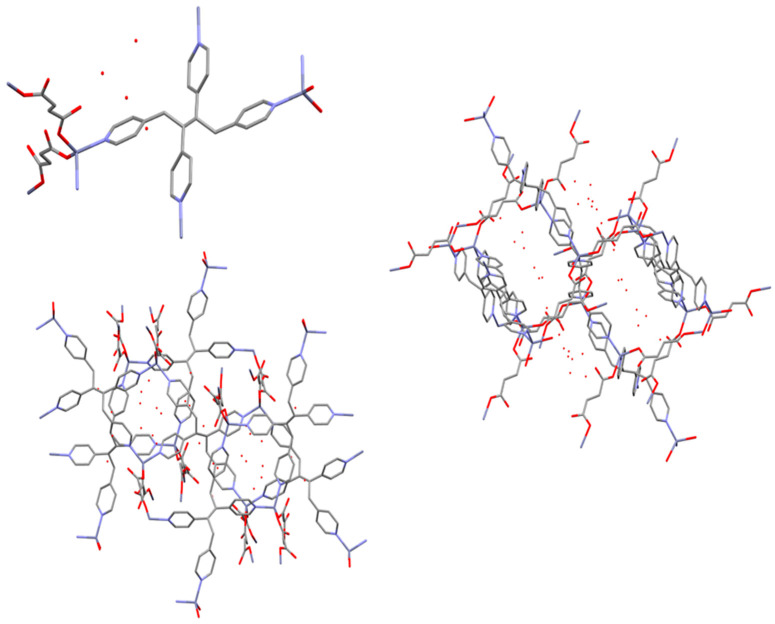
Molecular structure of [Zn(tpb)(fu)]·2H_2_O and 3D network [67]. (Zn, N, O).

Another representative of MOFs is [Zn(tpb)(fu)]·2H_2_O, whose 1D polymers (Figure 5) are bridged by a monodentate fumaric acid anion to form a 3D network (Figure 5) [67]. The tpb ligand is tetradentate and coordinates to four different zinc cations. The complex shows an intense fluorescence band (422 nm); it is not MLCT and LMCT charge transfer, but it is likely to be *π*–π* fluorescence emission. This type of emission is explained as enhanced emission of the tpb ligand when the ligand (tpb) is coordinated to zinc, and this circumstance strengthens the conformational rigidity of the tpb ligand. Similar emissions and the situations that cause these emissions are described for other coordination compounds such as [Zn(H_2_O)_2_(dbipy)(fu)] and [Zn(fu)(bpmp)(H_2_O)_2_] [49,51].

**Figure 6:**
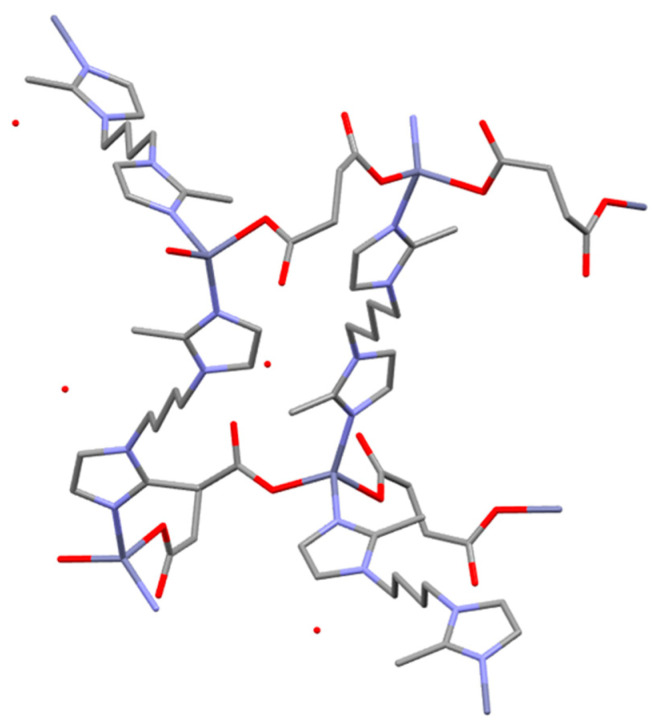
The network of the complex {[Zn(bib)(fu)]·CH_3_OH}*_n_* [104]. (Zn, N, O).

For the coordination polymer {[Zn(bib)(fu)]·CH_3_OH}*_n_*, a diamond-type network is formed (Figure 6), which is composed of two monodentate-coordinated fu^2−^ and two 1,4-bis(2-methyl-imidazol-1-yl)butane (bib) ligands, for which the coordination mode is *syn-anti*. The bridge ligand bib, which coordinates to zinc cations {Zn-bib-Zn-bib-Zn}*_n_*, forms a 1D chain (*meso-helix*). Similarly, a 1D polymer {Zn-fu-Zn-fu-Zn}*_n_* is formed, which is *meso-helical*, where the bridge is a fumaric acid anion. The two 1D polymers are constructed along different axes, which are connected to each other to form a 3D diamond-like rhombic structure. The 3D network contains potential voids. The equivalent diamond networks, which are independent, interpenetrate each other (as a quadruple interpenetrating topology) [104].

### 3.3. Nickel

#### 3.3.1. Nickel with 2,2′-Thiodiacetic Acid and N Donor Ligands

For the coordination compounds of nickel with 2,2′-thiodiacetic acid and *N* donor ligands, the number is equal to seven structurally characterized compounds, which are listed together with their coordination polyhedra and donor sets in Table 8.

[Ni(tda)(1,3-pn)(H_2_O)]·H_2_O and [Ni_2_(μ-tda)_2_(1,2-pn)_2_] evaluated against *E. faecalis*, *S. aureus, P. aeruginosa*, *E. coli* bacteria showed MIC 8.44, 8.44, 33.755, and 8.44 mg/L, and 8.44, >135, >135, 67.50 mg/L, respectively. The lower antibacterial activity of the binuclear complex can be due to its lower stability in solution.

Similarly to copper and zinc, complexes with tda chelating mode were proved with monodentate or bidentate N ligands. The complexes are mononuclear with the exception of en and 1,2-pn complexes where tda serve as bridges to nickel central atoms too. Perchlorate nickel pmdien complex contains bridging tda without S coordination.

#### 3.3.2. Nickel with 3,3′-Thiodipropionic Acid/3,3′-Dithiodipropionic Acids and N Donor Ligands

Compounds of nickel with 3,3′-thiodipropionic acid and 3,3′-dithiodipropionic acid with *N* donor ligands have not yet been structurally characterized.

#### 3.3.3. Nickel with Fumaric Acid and N Donor Ligands

More than a dozen Ni(II) compounds with fumaric acid, and *N* donor ligands have been prepared and structurally characterized so far, a list of which is given in Table 9.

As a potential mimicking metalloenzyme, the complex [Ni_2_(*μ-*bpym)(fu)_2_(H_2_O)_6_]·5H_2_O could be used in the future, whose molecular structure includes five water molecules crystal-bound and packed to form a 1D chain that is stabilized by hydrogen bonds [69]. The elimination of the water molecules starts at 30 °C, and ends at 224 °C, when both the crystal-bound and coordinated water molecules are lost in a few steps. The greatest changes occur at the crystal-to-crystal phase transitions between 45 and 55 °C, and it is in this interval that the loss of the water chain occurs. The crystalline phase disappears at 150 °C. The amorphous substance is further thermally stable up to 305 °C. For this coordination compound, a cycle of dehydration and rehydration is possible, which may be repeated. [Ni_2_(*μ-*bpym)(fu)_2_(H_2_O)_6_]·5H_2_O shows fluorescence emission at 395.8 nm, which is due to ligand emission and simultaneous ligand–metal charge transfer. The complex was further studied for magnetic properties where antiferromagnetic interaction was observed [69].

Another possible application of nickel–fumaric acid complexes is as an accelerator of the thermal decomposition of ammonium perchlorate and also as a promoter of solid fuel combustion for the complex [Ni_3_(Hdatrz)_6_(fu)_2_(H_2_O)_4_]fu. This trinuclear nickel coordination compound has been prepared by the dehydration of [Ni_3_(Hdatrz)_6_(fu)_2_(H_2_O)_4_]fu·11H_2_ O under a nitrogen atmosphere at 225 °C. The effect of accelerating the decomposition of ammonium perchlorate is due to the thermal decomposition of [Ni_3_(Hdatrz)_6_(fu)_2_(H_2_O)_4_]fu, which results in the release of large amounts of heat (up to 659.3 kJ·mol^−1^) and nickel oxide [110].

A similar 3D network formed by ligand–metal bonds, as in the case of the {[Zn(bib)(fu)]·CH_3_OH}*_n_* complex, was also found for the nickel coordination compound [Ni(fu)(bpe)], where the ligand 1,2-bis(4-pyridyl)ethane is in a *trans-*conformation. The [Ni(fu)(bpe)] network joins to form a five-stranded diamond-type permeating chain where adamantane-like units can be observed. The cycles in the adamant-type unit vary in bond length between the nickel cation and the carboxylate group and the nickel cation and the bpe ligand. A similar ligand, 1,3-bis(4-pyridyl)propane, was used in the synthesis of the complex [Ni(fu)(bpp)(H_2_O)] [107]. However, the ligand in the complexes has different conformations, is quite flexible and the N–N distance is, therefore, different (3.9–10.1 Å) [111], whereas in the complex [Ni(fu)(bpp)(H_2_O)], the N–N distance is 8.58 Å. The individual layers of the 2D network are wavy due to the *trans-conformation of* 1,3-bis(4-pyridyl)propane, which protrudes out of the plane. Both of these nickel complexes exhibit a weak fumarate-mediated antiferromagnetic interaction between the nickel ions in the 1D polymers [107].

The nickel complex with fumaric acid and the *N* donor ligand, [Ni(*μ-fu*)(py)_3_]·py·2H_2_O, is hygroscopic. The solvent molecules are adsorbed in micropores from which the molecules are eliminated by increasing the temperature, which is accompanied by a color change (from blue to light blue). The 2D polymer also has adsorption properties, but they are not significant compared to other similar complexes (e.g., [Cu(tp)(py)_2_(H_2_O)]·py·2H_2_O) due to the existence of the so-called zero-dimensional micropores where the nitrogen molecules are adsorbed. While other nickel complexes with dicarboxylic acids and *N* donor ligands exhibit antiferromagnetic properties, [Ni(*μ-*fu)(py)_3_]·py·2H_2_O exhibits paramagnetic properties [109].

Fumarate complexes of Cu, Zn, and Ni are mostly polymers. Binuclear complexes are formed in cases of monodentate or bidentate N ligands only. Moreover, counter ions such as nitrates and perchlorates probably stabilize lower nuclearity. Plenty of fumarate complexes have been prepared with the aim of higher nuclearity and MOF formation. That is why polydentate N donor ligands, which can also serve as bridges, were used. In cases of polymers and MOFs, limited solubility is expected.

## 4. Conclusions

The aim of the review was to perform a literature search of the current state of the art of chemistry of coordination compounds of copper, zinc, and nickel involving four dicarboxylic acids (2,2′-thiodioacetic acid, 3,3′-thiodipropionic acid, 3,3′-dithiodipropionic acid, and fumaric acid) and *N* donor ligands. Their coordination possibilities are discussed together with some interesting physical properties and potential biological activities are given in Table 10.

Although many complexes have been prepared and their structures solved, biological behavior has been studied rather seldom. It is obvious that studies of biological properties are limited by the solubility and stability of complexes in water solution. The solution stability of copper and nickel were studied by electron and fluorescence spectroscopies, and cyclic voltammetry. The complexes listed in Table 10 were mostly studied for their antibacterial properties. Bidentate ligand phen, used in the preparation of the complexes, is well known for its biological influence and intercalating binding to DNA. The structure of complexes plays a very important role in binding to DNA, where, for example, [Cu_2_(pmdien)_2_)(H_2_O)_2_(*μ*-tdp)](ClO_4_)_2_ forms a helical structure with hydrogen bonds. Antibacterial properties were reported for complexes with tridentate pmdien, as well as for complexes with bidentate diamines. Although zinc complexes with dicarboxylate acids were prepared as polymers or MOFs, they can find applications as drug carriers and are prospective from this point of view. Especially in times when resistance of some bacteria to antibiotics is growing, complexes with mixed donor ligands can be a way of overcoming the resistance. Our knowledge of the biological activities of already known complexes and their new synthesized analogs should be extended.

## Figures and Tables

**Table 1:** Summary of structurally characterized coordination compounds of copper with 2,2′-thiodiacetic acid and *N* donor ligands. For the donor atoms, the lower cursor indicates the ligands of which they are a part.

Compound	Coordinating Polyhedron	Donor Set	Literature
[Cu(tda)(phen)]_2_·H_2_tda	Defor. OC-6	*S_tda_, O_tda(i)_, OO_tda(ii)_, NN_phen_*	[74]
[Cu(tda)(im)_2_(H_2_O)]	Defor. OC-6	*S_tda_, O_tda(i)_, OO_tda(ii)_, NN_im_*	[47]
[Cu(tda)(5Mphen)]·2H_2_O	SPY-5	*S_tda_, OO_tda_, NN_5Mphen_*	[47]
[Cu(bipy)(tda)(H_2_O)]·4H_2_O	Defor. TBPY-5	*S_tda_, OO_tda_, NN_bipy_*	[38]
[Cu(terpy)(tda)]*_n_*	Defor. TBPY-5	*O_tda(i)_, O_tda(ii)_, NNN_terpy_*	[38]
[(phen)_2_Cu(*μ-*tda)Cu(phen)] (NO_3_)_2_ ·5H_2_O	Cu(1) Defor. SPY-5, Cu(2) TBPY-5—SPY-5	*Cu(1) O_tda_, NN_phen(i)_, NN_phen(ii)_, Cu(2) S_tda_, OO_tda_, NN_phen(iii)_*	[8]
[(H_2_O)(pmdien)Cu(*μ-*tda)Cu(pmdien) (H_2_O)] (ClO_4_)_2_	Defor. SPY-5	*O_tda_, O_H2O_, NNN_pmdien_*	[8]
[Cu(tmen)(tda)]·(H_2_tmen) (NO_3_)_2_·H_2_O	SPY-5	*OOS_tda_, NN_tmen_*	[57]
[Cu_4_(tpbn)_2_(tda)_2_(H_2_O)_4_](ClO_4_)_4_	Defor. SPY-5	*O_tda_, O_H2O_, NNN_tpbn_*	[75]
[Cu_4_(tpbn)_2_(tda)_2_(H_2_O)_4_](ClO_4_)_4_·4H_2_ O	Defor. SPY-5	*O_tda_, O_H2O_, NNN_tpbn_*	[75]
[(phen)_2_Cu(*μ-*tda)Cu(phen)] (ClO_4_)_2_·1.5H_2_O	Cu(1) TBPY-5, Cu(2) SPY-5	*Cu(1) OOS_tda_, NN_phen_, Cu(2) S_tda_, O_tda_, NN_phen(ii)_, NN_phen(ii)_*	[73]
[Cu(phen)(tda)]·2H_2_O	SPY-5	*OOS_tda_, NN_phen_*	[73]

**Table 2:** Summary of structurally characterized copper complexes with 3,3′-thiodipropionic acid and *N* donor ligands.

Compound	Coordinating Polyhedron	Donor Set	Literature
{[Cu(*μ*_3_-tdp)(phen)]·2H_2_O}*_n_*	Defor. SPY-5	*O_tdp(i),_ OO_tdp(ii)_, NN_phen_*	[78]
{[Cu(*μ*_3_-tdp)(bipy)]·H_2_O}*_n_*	SPY-5	*O_tdp(i)_, O_tdp(ii)_, O_tdp(iii)_, NN_bipy_*	[78]
[Cu_2_(*μ*_2_-tdp)(phen)_4_](NO_3_)_2_·2H_2_O	Defor. OC-6	*OO_tdp_, NN_phen(i)_, NN_phen(ii)_*	[78]
[Cu(tdp)(H_2_O)(bim)_3_]·4H_2_O	Defor. OC-6	*O_tdp_, O_H2O_, N_bim(i)_, N_bim(ii)_ N_bim(iii)_*	[79]
{[Cu(μ_2_-tdp)(bim)_2_]·4H_2_O}*_n_*	Defor. OC-6	*OOtdp_(i)_, OOtdp_(ii)_, Nbim_(i),_ Nbim_(ii)_*	[79]
[Cu(*μ*_3_-tdp)(im)_2_]*_n_*	Defor. SPY-5	*Otdp_(i)_, O_tdp(ii)_, Otdp_(iii)_, N_im(i)_, Nim_(ii)_*	[46]
{[Cu(*μ*_3_-tdp)(1-mim)_2_]·0.5H_2_O}*_n_*	SPY-5	*Otdp_(i)_, Otdp_(ii)_, Otdp_(iii)_, N1-mim_(i),_ N1-mim_(ii)_*	[46]
{[Cu_2_(*μ*_3_-tdp)_2_(4-mim)_4_]·H_2_O}*_n_*	SPY-5	*Otdp_(i)_, Otdp_(ii)_, Otdp_(iii)_, N4-mim_(i),_ N4-mim_(ii)_*	[46]
[Cu_2_(pmdien)_2_(H_2_O)_2_(*μ*-tdp)](ClO_4_)_2_·H_2_O	TBPY-5	*O_tdp_, O_H2O_, NNNpmdien*	[53]
{[Cu_2_(tdp)_4_(QX)]*_n_*·DMF}*_n_*	OC-6	*Cu, Otdp_(i)_, Otdp_(ii)_, Otdp_(iii)_, Otdp_(iv)_, N_QX_*	[80]

**Table 3:** Structurally characterized copper complexes with 3,3′-dithiodipropionic acid and *N* donor ligands.

Compound	Coordinating Polyhedron	Donor Set	Literature
[Cu(dpa)(dtdp)]*_n_*	Defor. OC-6	*OO_dtdp(i)_, OO_dtdp(ii)_, NN_dpa_*	[59]
[{(phen)Cu}_2_(*μ*-dtdp)_2_]·2H_2_O	SP-4	*OO_dtdp_, NN_phen_*	[81]
[Cu_2_(*μ*-dtdp)(pmdien)_2_(H_2_O)_2_](ClO_4_)_2_	SPY-5	*O_dtdp_, O_H2O_, NNN_pmdien_*	[82]

**Table 4:** List of structurally characterized coordination compounds of copper with fumaric acid and *N* donor ligands.

Compound	Coordinating Polyhedron	Donor Set	Literature
[Cu_2_(dpypda)_2_(fu)_2_]*_n_*·8*n*H_2_O	Defor. OC-6	*Cu, O_fu(i)_, _Ofu(ii)_, NNN_dpypda_*	[84]
[Cu(im)_2_(H_2_O)(fu)]*_n_*	SPY-5	*O_fu(i)_, _Ofu(ii)_, O_H2O_, N_im(i)_, N_im(ii)_*	[85]
[Cu_4_(ophen)_4_(fu)]	SPY-5	*Cu(1) O_fu_, O_ophen(i),_ NN_ophen(ii)_, Cu(2)*	[56]
[Cu_4_(obipy)_4_(fu)]·6H_2_O	Cu(1) SPY-5, Cu(2) SP-4	*Cu(1) O_fu_, O_obipy(i),_ NN_obipy(ii)_, Cu(2),* *Cu(2) O_obipy(iii)_, NN_obipy(iv)_, Cu(1)*	[56]
[Cu_2_(fu)(phen)_4_](ClO_4_)_2_ ·2H_2_O	Defor. OC-6	*OO_fu_, NN_phen(i)_, NN_phen(ii)_*	[43]
[Cu(fu)(phen)(H_2_O)]*_n_*	Defor. OC-6	*O_fu(i)_, OO_fu(iii)_, O_H2O_, NN_phen_*	[43]
[Cu_2_(fu)(bipy)_2_(H_2_O)_2_]*_n_*(ClO_4_)_2*n*_	SPY-5	*OO_fu(i)_, O_H2O_, NN_bipy_*	[43]
{[Cu_2_(L^4^)_2_(fu)]·(H_2_O)·(MeOH)}*_n_*	Defor. SPY-5	*OON_L4_, OO_fu_*	[86]
{[Cu(DMEDA)(*μ*-fu)(μ-H_2_O)]*_n_*	OC-6	*O_fu(i)_, _Ofu(ii)_, _OH2O(i)_, _OH2O(ii)_, NN_DMEDA_*	[52]
[Cu_2_(pmdien)_2_(*μ*-fu)(H_2_O)_2_](ClO_4_)_2_	SPY-5	*O_fu_, O_H2O_, NNN_pmdien_*	[45]
[Cu_2_(TPA)_2_(*μ*-fu)](ClO_4_)_2_·2H_2_O	TBPY-5	*O_fu_, NNNN_TPA_*	[45]
[Cu(fu)(pyphen)]*_n_*	Defor. TBPY-5	*O_fu(i)_, _Ofu(ii)_, _Ofu(iii)_, NN_pyphen_*	[87]
[Cu_2_(fu)_2_(bimb)_2_(H_2_O)_2_]*_n_*	SPY-5	*O_fu(i)_, _Ofu(ii)_, O_H2O_, NN_bimb_*	[44]
[Cu(dmbipy)(fu)(H_2_O)]*_n_*	OC-6	*OO_fu(i)_, _Ofu(ii)_, O_H2O_, NN_dmbipy_*	[88]
[Cu_2_(fu)(*μ*-OH)(bipy)_2_(H_2_O)]	SPY-5	*O_fu_, O_H2O_, O_OH_, NN_bipy_*	[89]
[Cu(fu)(tmen)]·2H_2_O	SP-4	*O_fu(i)_, _Ofu(ii)_, NN_tmen_*	[89]

**Table 5:** Structurally characterized zinc complexes with thiodiacetic acid and *N* donor ligands.

Compound	Coordinating Polyhedron	Donor Set	Literature
[Zn(bipy)(tda)(H_2_O)]·4H_2_O	OC-6	*OOS_tda_, O_H2O_, NN_bipy_*	[55]
[Zn(tda)(phen)]_2_·5H_2_O	Defor.OC-6	*OOS_tda(i)_, O_tda(ii)_, NN_phen_*	[63]
[Zn(bib)(tda)]*_n_*	Defor. T-4	*O_tda(i)_, O_tda(ii)_, N_bib(i)_, N_bib(ii)_*	[90]

**Table 6:** Summary of Zn(II) coordination compounds prepared and structurally characterized so far with 3,3′-thiodipropionic acid and *N* donor ligands.

Compound	Coordinating Polyhedron	Donor Set	Literature
[{Zn(phen)(H_2_O)}_2_(*μ*-tdp)_2_]·3H_2_O	Defor. OC-6	*O_tdp(i)_, OO_tdp(ii)_, O_H2O_, NN_phen_*	[92]
{[Zn(tdp)(bpe)]·2H_2_O}*_n_*	T-4	*O_tdp(i)_, O_tdp(ii)_, N_bpe(i)_, N_bpe(ii)_*	[61]
[Zn(tdp)(bpp)]*_n_*	T-4	*O_tdp(i)_, O_tdp(ii)_, N_bpp(i)_, N_bpp(ii)_*	[61]
[Zn(tdp)(bpypa)]*_n_*	T-4	*O_tdp(i)_, O_tdp(ii)_, N_bpypa(i)_, N_bpypa(ii)_*	[61]
[Zn(tdp)_2_(TMPy)_2_]*_n_*	SPY-5	*O_tdp(i)_, OO_tdp(ii)_, N_TMPy(i)_, N_TMPy(ii)_*	[91]
[Zn(tdp)(bipy)(H_2_O)]*_n_*	Defor. OC-6	*OO_tdp(i)_, O_tdp(ii)_, O_H2O_, NN_bipy_*	[62]

**Table 7:** Assigned coordination polyhedra and donor sets to structurally characterized zinc complexes with fumaric acid and *N* donor ligands.

Compound	Coordinating Polyhedron	Donor Set	Literature
[Zn(fu)(datrz)_2_]*_n_*	T-4	*O_fu_, N_datrz(i)_, N_datrz(ii)_, N_datrz(iii)_*	[93]
[Zn(fu)(bib)(H_2_O)]*_n_*	Defor.OC-6	*O_fu(i)_, O_fu(ii)_, O_H2O(i)_, O_H2O(ii)_, N_bib(i)_, N_bib(ii)_*	[94]
{[Zn(tptz)(fu)]·DMF}*_n_*	TBPY-5	*O_fu(i)_, O_fu(ii)_, NNN_tptz_*	[95]
{[Zn_2_(azbipy)_2_(fu)_2_]·H_2_O}*_n_*	SPY-5	*Zn(1) OO_fu(i)_, O_fu(ii)_, N_azbipy_, N_azbipy(ii)_, Zn(2) OO_fu(i)_, OO_fu(ii)_, N_azbipy_, N_azbipy(ii)_*	[96]
[Zn(tu)_2_(*μ*-fu)]	T-4	*O_fu(i)_, O_fu(ii)_, S_tu(i)_, S_tu(ii)_*	[97]
[Zn{tu(Me)_2_}_2_(fu)]*_n_*	T-4	*O_fu(i)_, O_fu(ii)_, S_tu(i)_, S_tu(ii)_*	[98]
[Zn(fu)(bpmp)(H_2_O)_2_]	OC-6	*O_fu(i)_, O_fu(ii)_, O_H2O(i)_, O_H2O(ii)_, N_bpmp(i)_, N_bpmp(ii)_*	[51]
[Zn(*μ*-fu)(L^5^)]*_n_*	SPY-5	*O_fu(i)_, O_fu(ii)_, NNN_L5_*	[64]
[Zn(fu)(pyphen)]*_n_*	Defor. TBPY-5	*O_fu(i)_, O_fu(ii)_, O_fu(iii)_, NN_pyphen_*	[99]
[Zn(tpb)(fu)]·2H_2_O	SP-4	*O_fu(i)_, O_fu(ii)_, N_tpb(i)_, N_tpb(ii)_*	[67]
[Zn(fu)(bbmi)_0,5_(H_2_O)]	SPY-5	*O_fu(i)_, O_fu(ii)_, O_fu(iii)_, O_H2O_, N_bbmi_*	[100]
[Zn_4_(OH)_2_(fu)_3_(4,4′-bipy)]	Zn(1) OC-6, Zn(2) T-4	*Zn(1) O_fu(i)_, OO_fu(ii)_, O_OH(i)_, O_OH(ii)_, N_4,4′-bipy_, Zn(2) O_fu(i)_, O_fu(ii)_, O_OH_, N_4,4′-bipy_*	[101]
[Zn(H_2_O)_2_(dbipy)(fu)]	Defor. TPR-6	*OO_fu(i)_, O_H2O(i)_, O_H2O(ii)_, NN_dbipy_*	[49]
{[Zn(fu)(3-pina)]·1.5H_2_O}*_n_*	T-4	*O_fu(i)_, O_fu(ii)_, N_3-pina(i)_, N_3-pina(ii)_*	[102]
[Zn(fu)(4-pna)]*_n_*	Defor. SPY-5	*OO_fu(i)_, O_fu(ii)_, N_4-pna(i)_, N_4-pna(ii)_*	[102]
[Zn(fu)(4-pina)]*_n_*	Defor. TBPY-5	*O_fu(i)_, O_fu(ii)_, O_fu(iii)_, N_4-pina(i)_, N_4-pina(ii)_*	[102]
[Zn_5_(pytpy)_8_(fu)_4_(H_2_O)_4_(OH)_2_]*_n_*·*n*(CH_3_OH)·*2n*(H_2_O)	Zn(1) and Zn(3) Defor. OC-6, Zn(2) Defor. TBPY-5	*Zn(1) a Zn(3) O_fu(i)_, O_fu(ii)_, O_fu(iii)_, O_fu(iv)_, N_pytpy(i)_, N_pytpy(ii)_, Zn(2) O_fu(i)_, O_fu(ii)_, O_fu(iii)_, N_pytpy(i)_, N_pytpy(ii)_*	[103]
[Zn(bpe)(fu)]*_n_*	T-4	*O_fu(i)_, O_fu(ii)_, N_bpe(i)_, N_bpe(ii)_*	[68]
{[Zn(bib)(fu)]·CH_3_OH}*_n_*	T-4	*O_fu(i)_, O_fu(ii)_, N_bib(i)_, N_bib(ii)_*	[104]
[Zn(phen(fu)]*_n_*	T-4	*O_fu(i)_, O_fu(ii)_, NN_phen_*	[105]

**Table 8:** Ni(II) complexes with 2,2′-thiodiacetic acid and *N* donor ligands that are structurally characterized.

Compound	Coordinating Polyhedron	Donor Set	Literature
[Ni(tda)(dabt)(H_2_O)]·H_2_O	Defor. OC-6	*OOS_tda_, O_H2O_, NN_dabt_*	[54]
[Ni(tda))(*1H*-im)_3_]·H_2_O	Defor. OC-6	*OOS_tda_, N_1H-im(i)_, N_1H-im(ii)_, N_1H-im(iii)_*	[72]
[Ni(bipy)(tda)(H_2_O)]·4H_2_O	Defor. OC-6	*OOS_tda_, O_H2O_, NN_bipy_*	[7]
[(en)Ni(*μ*-tda)_2_Ni(en)]·4H_2_O	Defor. OC-6	*OOS_tda_, NN_en_*	[7]
[Ni_2_(pmdien)_2_(H_2_O)_2_(*μ*-tda)](ClO_4_)_2_·H_2_O	Defor. OC-6	*OO_tda_, O_H2O_, NNN_pmdien_*	[41]
[Ni(tda)(1,3-pn)(H_2_O)]·H_2_O	Defor. OC-6	*OOS_tda_, O_H2O_, NN_1,3-pn_*	[82]
[Ni_2_(μ-tda)_2_(1,2-pn)_2_]	Defor. OC-6	*OOS_tda_, NN_1,2-pn_*	[82]

**Table 9:** Overview of nickel complexes prepared and structurally characterized with fumaric acid and *N* donor ligands.

Compound	Coordinating Polyhedron	Donor Set	Literature
[Ni(*μ*-fu)(4-Cnpy)_2_(H_2_O)_2_]*_n_*	OC-6	*O_fu(i)_, O_fu(ii)_, O_H2O(i)_, O_H2O(ii)_, N_4-Cnpy(i)_, N_4-Cnpy(ii)_*	[40]
[Ni(fu)(nam)_2_(H_2_O)_2_]*_n_*	Defor. OC-6	*O_fu(i)_, O_fu(ii)_, O_H2O(i)_, O_H2O(ii)_, N_nam(i)_, N_nam(ii)_*	[66]
[Ni(fu)(bpe)]	Defor. OC-6	*OO_fu(i)_, OO_fu(ii)_, N_bpe(i)_, N_bpe(ii)_*	[107]
[Ni(fu)(bpp)(H_2_O)]	Defor. OC-6	*OO_fu(i)_, O_fu(ii)_, O_H2O_, N_bpp(i)_, N_bpp(ii)_*	[107]
[Ni(fu)(bim)(H_2_O)_2_]	OC-6	*O_fu(i)_, O_fu(ii)_, O_H2O(i)_, O_H2O(ii)_, N_bim(i)_, N_bim(ii)_*	[108]
[Ni_2_(*μ-*fu)(phen)_4_(H_2_O)_2_] ·fu·16H_2_O	OC-6	*O_fu_, O_H2O_, NN_phen(i)_, NN_phenii)_*	[71]
[Ni_2_(ntb)_2_(*μ-*fu)(H_2_O)(CH_3_OH)] (NO_3_)_2_·6CH_3_OH·H_2_O	OC-6	*Ni(1) O_fu_, O_CH3OH_, NNNN_ntb(i)_, Ni(2) O_fu_, O_H2O,_ NNNN_ntb(ii)_*	[65]
[Ni(*μ-*fu)(py)_3_]·py·2H_2_O	OC-6	*OO_fu(i)_, O_fu(ii)_, N_py(i)_, N_py(ii)_, N_py(iii)_*	[109]
[Ni(fu)(dpa)(H_2_O)_2_·4H_2_O]*_n_*	Defor. OC-6	*O_fu(i)_, O_fu(ii)_, O_H2O(i)_, O_H2O(ii)_, NN_dpa_*	[39]
[Ni_2_(*μ-*bpym)(fu)_2_(H_2_O)_6_]·5H_2_O	Defor. OC-6	*O_fu_, O_H2O(i)_, O_H2O(ii)_, O_H2O(iii)_, NN_dpa_*	[69]
[Ni(fu)(phen)(H_2_O)]*_n_*	Defor. OC-6	*OO_fu(i)_, O_fu(ii)_, O_H2O(i)_, NN_phen_*	[70]
[Ni_3_(Hdatrz)_6_(fu)_2_(H_2_O)_4_]fu·11H_2_O	Ni(1), Ni(2) and Ni(3) Defor.OC-6	*Ni(1) a (3) O_fu(i)_, O_fu(ii)_, O_H2O(i)_, O_H2O(ii)_, NNN_Hdatrz_, Ni(2) N_Hdatrz(i)_, N_Hdatrz(ii)_, N_Hdatrz(iii)_, N_Hdatrz(iv)_, N_Hdatrz(v)_, N_Hdatrz(vi)_*	[110]
[Ni_2_(fu)(OH)_2_(dpa)_2_]·2H_2_O]*_n_*	Defor. SPY-5	*O_fu_, O_OH(i)_, O_OH(ii)_, NN_dpa_*	[42]

**Table 10:** Overview of complexes used in biological studies.

Compound	Biological Activity Studied	Stability in Solution	Literature
[(phen)_2_Cu(*μ-*tda)Cu(phen)](ClO_4_)_2_·1.5H_2_O	Inhibition zone, *B. subtilis*, *S. aureus*, *E. faecalis*, *E. coli*, *K. pneumoniae*, *P. aeruginosa*	UV-vis, CV	[73]
[Cu(phen)(tda)]·2H_2_O	Inhibition zone, *B. subtilis*, *S. aureus*, *E. faecalis*, *E. coli*, *K. pneumoniae*, *P. aeruginosa*	UV-vis, CV	[73]
[Cu_2_(pmdien)_2_(H_2_O)_2_(*μ*-tdp)](ClO_4_)_2_·H_2_O	*E. coli*, *S. aureus*, MRSA, MDA-MB-231, HBL-100, p53, DNA binding, ROS	UV-vis	[53]
[{(phen)Cu}_2_(*μ*-dtdp)_2_]·2H_2_O	photoinduced DNA cleavage activity, ROS, CT-DNA binding	UV-vis	[81]
[Cu_2_(*μ*-dtdp)(pmdien)_2_(H_2_O)_2_](ClO_4_)_2_	*E. faecalis*, *S. aureus*, *P. aeruginosa*, *E. coli*	UV-vis	[82]
{[Cu_2_(L^4^)_2_(fu)]·(H_2_O)·(MeOH)}*_n_*	BSA, HSA, CT-DNA binding	UV-vis, fluorescence spectroscopy	[86]
[Ni(tda)(1,3-pn)(H_2_O)]·H_2_O	*E. faecalis*, *S. aureus*, *P. aeruginosa*, *E. coli*	UV-vis	[82]
[Ni_2_(μ-tda)_2_(1,2-pn)_2_]	*E. faecalis*, *S. aureus*, *P. aeruginosa*, *E. coli*	UV-vis	[82]

## Data Availability

Not applicable.

## Abbreviations

1-mim—1-methylimidazole; 1,2-pn—1,2-propanediamine; 1,3-pn—1,3-propanediamine; 1,8-dan-1,8-diaminonaphthalene; *1H-im—1H-*imidazole; 3-pina—3-pyridylisonicotinamide; 4-CNpy—4-cyanopyridine; 4-mim—4-methylimidazole; 4-pina—4-pyridylisonicotinamide; 4-pna—4-pyridylnicotinamide; 4,4′-bipyridine—4,4′-bipyridine; 5Mphen—5-methyl-1,10-phenanthroline; ampy—2-(aminomethyl)-pyridine; Bcl-2—B-cell lymphoma 2; Bcl-_XL—_B-cell lymphoma-extra large; bib—1,4-bis(2-methyl-imidazol-1-yl)butane; bim—benzimidazole; bimb—1,4-bis(imidazol-1-yl-methylene)benzene; bipy—2,2′-bipyridine; bpe—*trans*-*1*,2-bis(4-pyridyl)ethane; bpmp—bis(4-pyridylmethyl)piperazine; bpp—1,3-bis(4-pyridyl)propane; bpypa—4,4′-bis(4-pyridyl)diphenylamine; bpym—2,2′-bipyrimidine; BSA—bovine serum albumin; dabt—2,2′-diamino-4,4′-bi-1,3-thiazole; dbipy—5,5′-dimethyl-2,2′-bipyridine; defor.—deformed; dmbipy—4,4′-dimethyl-2,2′-bipyridine; DMEDA—*N,N′-*dimethylethane-1,2-diamine; DMF—dimethylformamide; dpa—di-2-pyridylamine; dpq—dipyrido[3,2-d:2′3′-f]quinoxaline; dpypda—*N^2^*,*N^4^*-di(pyridin-2-yl)pyrimidine-2,4-diamine; en—ethylenediamine; H_2_dtdp—3,3′-dithiodipropionic acid; H_2_fu—fumaric acid; H_2_tda—2,2′-thiodiacetic acid; H_2_tdp—3,3′-thiodipropionic acid; H_2_tp—terephthalic acid; HBL-100—human breast epithelial cell line; Hdatrz—3,5-diamino-1,2,4-triazole; Hgly—glycine; HL^4^—(*E*)-2-((1-hydroxybutan-2-ylimino)methyl)phenol—product of condensation of salicylaldehyde with 2-amino-1-butanol; Hobipy—6-hydroxy-2,2′-bipyridine; Hophene—2-hydroxy-1,10-phenathroline; HSA—human serum albumin; im—imidazole; K_SV—_Stern-Volmer constant; L^1^—thiosemicarbazone; L^2^—1,10-phenanthroline or 2,2′-bipyridine; L^3^—*α-*aminoacidate, acetylacetonate or salicyl; L^5^—*N^1^*,*N^1^*-dimethyl-N*^2^*-(1-(pyridin-2-yl)ethylidene)ethane-1,2-diamine; MDA-MB-231 human breast adenocarcinoma cancer cell line; MIC—minimum inhibitory concentration; MOF—metal–organic framework; MT-1/2—metallothionein-1/2; MT-3—methallothionein-3; nphen—5-nitro-1,10-phenanthroline; ntb—tris(2-bezimidazolylmethyl)amine; OC-6—octahedron; phen—1,10-phenanthroline; pmdien—*N,N,N,N′,N″,N″-*pentamethyldiethylenetriamine; pyphen—pyrazino[2,3-f][1,10]phenanthroline; pytpy—4′-(4-pyridyl)3,2′:6′,3′′-terpyridine; QX—quinoxaline; ROS—reactive oxygen species; RSH—thiols; SOD—superoxide dismutase; SP-4—square-planar; SPY-5—square pyramid; T-4—tetrahedron; TBPY-5—trigonal bipyramid; terpy—2,2′:6′,2′′-terpyridyl; tmen—*N,N,N,N′,N′-*tetramethylene-1,2-diamine; TMPy—4,4-trimethylenedipyridine; TPA—tris(2-pyridylmethyl)amine; tpb—1,2,3,4-tetra-(4-pyridyl)-butane; tpbn—*N,N′,N″,N‴-*tetrakis(2-pyridylmethyl)-1,4-diaminobutane; TPR-6—trigonal prism; tu—thiourea; tu(Me)_2_—*N,N′-*dimethylthiourea; VEGF—vascular endothelial growth factor 2-thiobarbituric acid reactive substance.
